# Hybridization-Driven Herbivorous Adaptation in Fish: Morphological, Digestive, Transcriptome, and Microbial Evidence from a Hybrid of *Megalobrama amblycephala* (♀) × *Culter mongolicus* (♂)

**DOI:** 10.3390/ijms27114775

**Published:** 2026-05-26

**Authors:** Yan Li, Chiye Zhao, Mingli Liu, Chaoying Luo, Zheduo Xiong, Hong Chen, Haitao Zhong, Jiaqi Jiang, Xushuai Xin, Yuheng Wang, Chun Zhang, Chang Wu, Qizhi Liu, Yu Sun, Shi Wang, Ming Wen, Fangzhou Hu, Shaojun Liu

**Affiliations:** 1 Engineering Research Center of Polyploid Fish Reproduction and Breeding of the State Education Ministry, College of Life Sciences, Hunan Normal University, Changsha 410081, China; 2 Yuelushan Laboratory, Changsha 410082, China

**Keywords:** feeding organ, digestive enzyme activity, intestinal microflora, transcriptome

## Abstract

Modifying the feeding habits of economically valuable carnivorous fish species towards omnivorous or herbivorous diets is of significant importance in aquaculture. In previous studies, we obtained a hybrid fish (BM) by crossing herbivorous female *Megalobrama amblycephala* (BSB) (♀) with carnivorous male *Culter mongolicus* (MC) (♂). Preliminary research indicated that BM exhibits herbivorous tendencies and rapid growth. To further evaluate the feeding characteristics and application potential of BM, this study systematically analyzed and compared BM with its parental groups, focusing on the structural traits of feeding organs, digestive enzyme activity, hepatic transcriptome, and gut microbiota features. The results demonstrate that BM possesses intermediate morphological traits in its feeding organs, with measurable ratios lying between those of BSB and MC and closer to BSB. In terms of intestinal morphology, BM also exhibits hybrid characteristics, showing greater similarity to BSB. Compared to BSB, BM exhibited significantly higher trypsin and lipase activities in both the intestine and liver (*p* < 0.05), although these levels remained lower than those in MC (*p* < 0.05) and were closer to BSB. The α-amylase activity in BM was significantly lower than in BSB (*p* < 0.05) but higher than in MC (*p* < 0.05). Regarding muscle composition, BM showed a significant increase in protein content compared to both parental lines BSB and MC (*p* < 0.05), while its crude fat content was significantly lower than that of the paternal line MC (*p* < 0.05), and showed no significant difference from the maternal line BSB. Transcriptome analysis revealed that differentially expressed genes in the liver of BM were significantly enriched in pathways related to nutrient intake and metabolism, including the MAPK signaling pathway, insulin signaling pathway, glycerophospholipid metabolism, adipocytokine signaling pathway, arginine and proline metabolism, and glycolysis/gluconeogenesis, all closely associated with feeding habits in fish. The analysis of gut microbiota showed greater similarity between BM and BSB. Overall, the findings demonstrate that BM is a high-quality hybrid fish with herbivorous tendencies and elevated muscle protein content, which highlights its considerable potential for reducing feed costs and promoting sustainable aquaculture. These results provide supporting data for the future promotion and utilization of BM.

## 1. Introduction

The feeding habits of fish not only influence their growth and development but also determine the production costs and economic benefits of aquaculture [1]. Fish feeding habits are primarily categorized into three types: carnivorous, herbivorous, and omnivorous. Throughout the long process of evolution, the feeding habits of fish have co-evolved in coordination with their feeding and digestive organs. For example, the mouth gapes of carnivorous fish are significantly larger than those of herbivorous fish [2]. Carnivorous fish exhibit higher levels of protease and lipase, while herbivorous fish possess higher levels of amylase and cellulase, which are crucial for breaking down carbohydrates and cellulose in plant-based foods [3,4].

The fish intestine is the primary organ for digestion and absorption, and its structure is adapted to the type of food consumed. The digestive tract is generally divided into three segments: the anterior intestine, middle intestine and posterior intestine, which differ in their absorptive and digestive functions [5]. In stomachless fish, the foregut is enlarged and serves as a food storage chamber, while the midgut and hindgut gradually narrow [6,7]. Generally, herbivorous fish have evolved longer intestines to increase food retention time, thereby enhancing the digestion and absorption efficiency of plant-based materials that are difficult to break down quickly [8,9]. The relative intestine length (RIL = intestine length/body length) of herbivorous fish typically ranges from 2 to 5, with grass carp being a representative example, having an RIL of 2.13 [10]. In contrast, carnivorous fish, which consume easily digestible food, have shorter and thicker intestines, usually with an RIL of less than 1. For instance, the carnivorous topmouth culter (*Culter alburnus*) studied by Liu et al. [11] has an RIL of 0.93, while studies by Pan et al. [12] reported RIL values of 0.66 for northern snakehead (*Channa argus*), 0.62 for mandarin fish (*Siniperca chuatsi*), and 0.74 for largemouth bass (*Micropterus salmoides*), all of which are below 1. Omnivorous fish exhibit intermediate RIL values between those of herbivorous and carnivorous species. Numerous researchers have studied fish intestinal RIL to predict dietary characteristics, highlighting RIL as a key indicator for evaluating fish feeding habits. Microorganisms are widely present in the intestines of fish and typically participate in the digestion of nutrients, which helps improve the metabolic capacity of fish [13,14]. Relevant studies have found that there are significant differences in the intestinal microbiota between carnivorous and herbivorous fish, confirming that the diversity of microbial communities is closely related to the feeding habits of fish [15,16].

Aquaculture is playing an increasingly important role in the fisheries industry, accounting for over 57% of the total production of aquatic animal products directly consumed by humans [17]. China is a leading aquaculture country, with its freshwater fish farming output ranking first globally. Among the major cultured fish species in China, a significant proportion are carnivorous fish, such as mandarin fish, largemouth bass, snakehead, and topmouth culter [18]. Generally, the natural food supply for carnivorous fish is limited, and large-scale aquaculture requires domestication through artificial feeding. Furthermore, high-protein feed for carnivorous fish typically contains 45–50% crude protein, costing 30–40% more than herbivorous feed (15–25% protein). Additionally, nitrogen waste from high-protein diets increases ammonia levels, requiring more intensive water management [19]. Therefore, research aimed at modifying the feeding habits of carnivorous fish, expanding their food sources, and achieving omnivorous or herbivorous diets will contribute to the sustainable and healthy development of the aquaculture industry.

Currently, research on modifying the feeding habits of carnivorous fish focuses on two main approaches. The first involves optimizing domestication conditions and feed formulations to enable carnivorous fish to consistently accept formulated feeds. For example, largemouth bass, a typical carnivorous species, has a relatively mature domestication process that ensures successful adaptation to formulated feeds, effectively lowering production costs [20]. Additionally, carnivorous fish such as mandarin fish [21], northern snakehead [22], and yellowcheek carp (*Elopichthys bambusa*) [23] have all been successfully domesticated to consume formulated feeds. The second is genetic improvement, which alters the fish’s genetic makeup to achieve heritable changes in feeding preference. Among genetic improvement strategies, hybridization is one of the most widely applied methods. By crossing carnivorous with herbivorous or omnivorous species, hybrid offspring can exhibit shifted feeding habits, often toward omnivory or herbivory. For example, Zhong et al. [24] crossed carnivorous largemouth bass with omnivorous green sunfish and obtained hybrids with clear omnivorous tendencies. Li et al. [11] studied hybrids of herbivorous blunt snout bream (*Megalobrama amblycephala*) and carnivorous topmouth culter, finding that the hybrids displayed herbivore-like intestinal and microbial traits. Wang et al. [25] reported that hybrids between herbivorous and carnivorous *Schizothorax* species exhibited omnivorous feeding behavior. These studies demonstrate that hybridization alters the genetic architecture of the offspring, leading to changes in physiological and metagenomic traits that underpin feeding habit shifts. Importantly, hybridization is not only a research tool but also a feasible and sustainable solution for aquaculture. Its reproducibility is ensured by the use of purebred parental lines that can be maintained and crossed repeatedly to produce consistent F_1_ hybrids. Many hybrid offspring are fertile, allowing further propagation through self-crossing or backcrossing to establish stable populations. Moreover, artificial hybridization techniques are well-established in aquaculture, enabling large-scale commercial application. Unlike domestication, which often requires multiple generations of selection, hybridization can produce desired feeding traits in the F1 generation, significantly shortening the breeding cycle. Therefore, hybridization has general applicability for modifying feeding habits across various fish species, providing a practical and reproducible genetic strategy for sustainable aquaculture.

In our previous studies, a viable population of hybrid fish (BM) was successfully produced by crossing herbivorous female *Megalobrama amblycephala* (BSB) (♀) with carnivorous male *Culter mongolicus* (MC) (♂). Preliminary findings suggested that BM exhibits favorable traits, including herbivorous feeding inclination and rapid growth [18]. To further evaluate the feeding characteristics and potential application of BM, this study systematically compared BM with its parental species in terms of feeding apparatus morphology, muscle nutritional composition, digestive enzyme activity, hepatic transcriptome, and gut microbiota structure.

## 2. Results

### 2.1. Structural Characteristics of the Head and Mouth

The structural characteristics of the head and mouth in BSB, MC, and BM are shown in Table 1. Compared with BSB and MC, BM exhibited a shorter head, a slightly upturned mandible, a moderate gape, and a slightly more developed tongue bone. Regarding the quantitative traits of feeding organs, BM showed significantly smaller MW/MH, HL/HH, and MH/HH ratios than MC (*p* < 0.05), while these values were significantly larger than those of BSB (*p* < 0.05). In addition, BM had a significantly larger BL/MW ratio compared to MC (*p* < 0.05), but a significantly smaller ratio compared to BSB (*p* < 0.05).

### 2.2. Structural Characteristics of the Intestine

**Figure 1:**
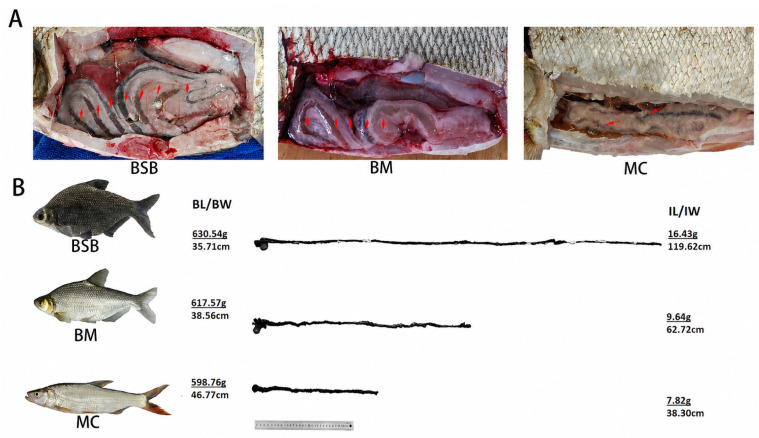
Gut structures of hybrid fish and their parents. (**A**) Gut structures of the BSB and MC parents and the BM hybrids; (**B**) Relative gut lengths of the BSB and MC parents and the BM hybrids; both ends showed the fish phenotype, body length and body weight, gut length and gut weight of fish individuals; bar = 20 cm. Red arrows showed the intestinal-loop times.

**Figure 2:**
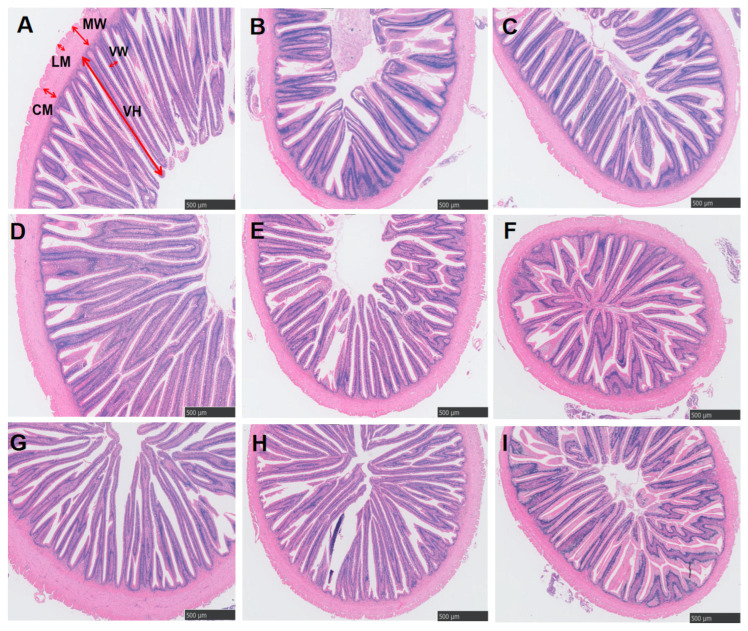
Intestinal histology of BSB (**A**–**C**), BM (**D**–**F**), and MC (**G**–**I**). Columns: anterior, middle, and posterior intestine. MW: wall thickness; CM: circular muscle; LM: longitudinal muscle; VH: mucosal fold height; VW: mucosal fold width. Scale bar = 500 μm.

As shown in Figure 1A, the three fish species exhibited marked differences in the gross anatomical arrangement of the intestine within the abdominal cavity. Here, “intestinal folds” refers to the number of visible bends or coils of the intestine, which can be directly observed upon dissection. MC exhibited the fewest intestinal folds (2–3), BSB had the most (7–8), and BM displayed an intermediate number (5–6). Notably, BM more closely resembled its maternal parent BSB, in having a greater number of intestinal folds. Additionally, the herbivorous BSB showed the longest intestinal structure, while the carnivorous MC had the shortest, with the intestinal length of the BM hybrids being intermediate between the two parents (Figure 1B). Analysis of intestinal traits among the parental fish and their hybrids indicated that the relative gut length (RGL) of BM (1.90) was intermediate between that of BSB (2.24) and MC (0.97) (Table 1). Overall, these findings confirm that the intestinal length and fold number of BM lie between those of the parental species, aligning with its intermediate herbivorous adaptation. Histological sections of the intestine revealed that BM and its parental species exhibited a similar morphological pattern, characterized by a thick anterior intestine and slender middle and posterior intestines. This pattern suggests that the anterior intestine can accommodate a larger volume of food, functioning analogously to the stomach in species that possess one. Regarding intestinal wall thickness (Figure 2), BM and its parents showed a gradual thinning from the anterior to the posterior intestine. Moreover, BSB had a significantly thinner intestinal muscular layer (Figure 2A–C) than MC (Figure 2G–I), while the BM hybrid displayed an intestinal wall thickness similar to its maternal parent BSB (Figure 2D–F). These findings further support the herbivorous tendency of BM in terms of feeding adaptation.

The results of measurements on mucosal folds height (VH), mucosal folds width (VW), intestinal wall thickness (MV), and the thickness of the circular muscle (CM) and longitudinal muscle (LM) layers, based on histological microstructural analysis of intestinal tissue sections, are presented in Table 2. Significant variations were observed among the three fish species in terms of villus length and width, as well as the thickness of the intestinal muscle layers. BSB exhibited a thicker intestinal wall with large, broad, leaf-shaped intestinal villi. In contrast, MC displayed smaller intestinal villi, a thinner intestinal wall, and well-developed muscle layers (Figure 2).

### 2.3. Digestive Enzyme Activity

**Figure 3:**
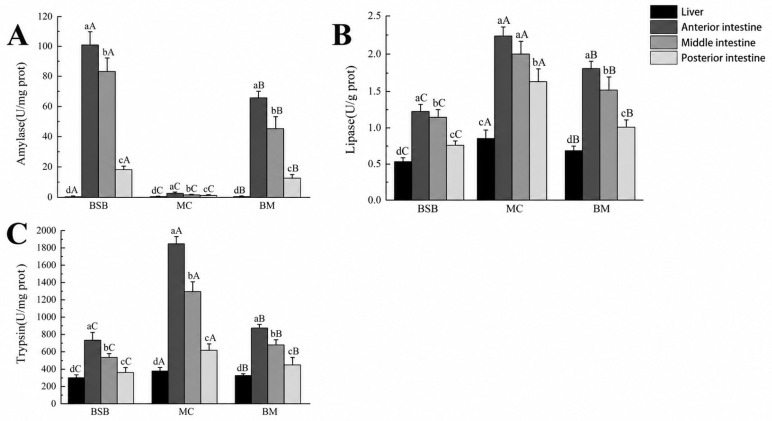
The activities of α-amylase, lipase, and trypsin in MC, BSB, and BM. (**A**) The activity of α-amylase; (**B**) The activity of lipase; (**C**) The activity of trypsin. Different lowercase letters within the same fish species indicate significant differences (*p* < 0.05), while different uppercase letters among different fish species indicate significant differences (*p* < 0.05).

The activities of major digestive enzymes in the liver and intestine of the three experimental fish groups are shown in Figure 3. Within the same tissue types, α-amylase activity in BM was significantly lower than that in BSB (*p* < 0.05) and significantly higher than that in MC (*p* < 0.05) (Figure 3A). Meanwhile, the activities of trypsin and lipase in BM were significantly lower than those in MC (*p* < 0.05) and significantly higher than those in BSB (*p* < 0.05) (Figure 3B). In different tissues of the same species, enzyme activity followed a descending order from the anterior intestine to the middle intestine, posterior intestine, and liver (*p* < 0.05) (Figure 3C).

### 2.4. Proximate Composition Analysis

The proximate composition of the dorsal muscle is presented in Table 3. BM showed a crude protein content of 19.63%, which was significantly higher (*p* < 0.05) than that of both MC (18.3%) and BSB (18.5%). In contrast, the crude fat content of BM (1.6%) was significantly lower (*p* < 0.05) than that of MC (2.33%) but did not differ significantly (*p* > 0.05) from that of BSB (0.73%). The moisture content of BM (77.3%) was significantly lower than that of BSB (78.9%) (*p* < 0.05), while no significant difference (*p* > 0.05) was observed compared with MC (77.8%). Ash content did not differ significantly among BSB (1.13%), MC (1.0%), and BM (1.1%).

### 2.5. PCA and Differential Expression Analysis

**Figure 4:**
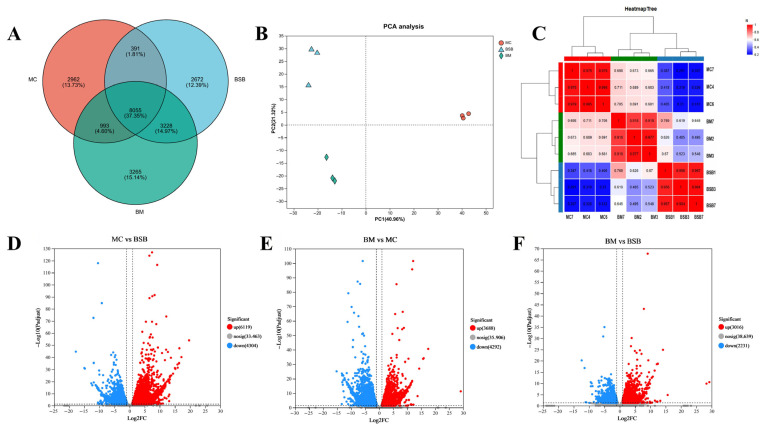
Analysis diagram of sample expression levels and KEGG enrichment of differentially expressed genes. (**A**) Venn diagram of gene expression levels of samples; (**B**) Principal component analysis (PCA) of samples; (**C**) Correlation analysis of samples; (**D**–**F**) Volcano plots analyses of differentially expressed genes in MC vs. BSB, BM vs. MC, and BM vs. BSB.

Following quality control, a total of 43,886 unigenes were identified (Table A2). The proportion of co-expressed genes between BM and MC (41.95%) was significantly lower than that between BM and BSB (52.32%) (*p* < 0.05) (Figure 4A). Principal component analysis (PCA) analysis showed that PC1 and PC2 explained 40.96% and 21.32% of the total variance, respectively, effectively distinguishing the three fish groups across all unigenes (Figure 4B). PCA was performed using the expression profiles of all unigenes from nine samples (three biological replicates for each group). Correlation analysis showed high intra-group correlations (Pearson’s r > 0.85 for all groups). Inter-group correlations between BM and BSB (r = 0.68) were higher than between BM and MC (r = 0.45), suggesting greater similarity in gene expression between BM and the maternal parent BSB. (Figure 4C). In the MC vs. BSB comparison, 10,423 differentially expressed genes (DEGs) were identified, of which 6119 were upregulated and 4304 were downregulated. For the BM vs. MC comparison, 7980 DEGs were detected, comprising 3688 upregulated and 4292 downregulated genes. For the BM vs. BSB comparison, 5247 DEGs were detected, comprising 3016 upregulated and 2231 downregulated genes (Figure 4D–F). Expression bias analysis revealed that the proportion of genes biased toward BSB in BM (68.20%) was significantly higher than that biased toward MC (31.79%). KEGG enrichment analysis of DEGs results show that most enriched pathways were related to nutrient-related metabolic signaling, including the metabolism of threonine, aspartate, and glutamate; glycine, arginine, and sulfur-containing amino acids; fatty acid degradation; protein processing in the endoplasmic reticulum; the PPAR pathway; biosynthesis of unsaturated fatty acids; and β-alanine metabolism. These pathways are closely linked to differences in nutrient intake and metabolism among the fish, reflecting their distinct feeding habits.

### 2.6. Gut Microbiota Characteristics

**Figure 5:**
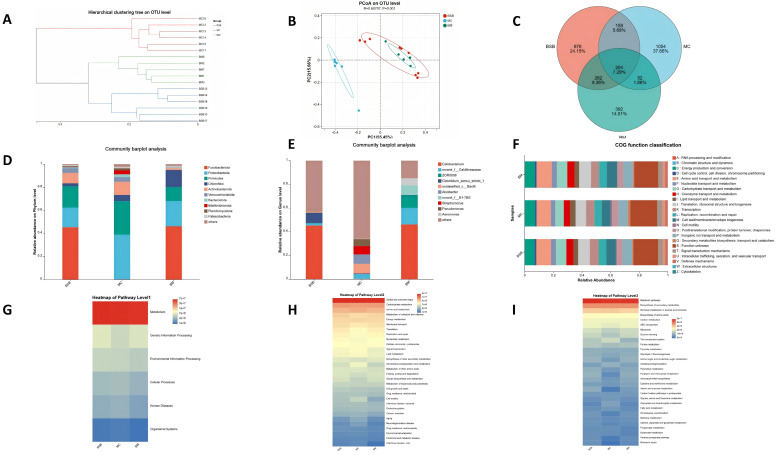
Characteristics of gut microbiota among BSB, MC, and BM. (**A**) Hierarchical clustering dendrogram of MC, BM, and BSB samples; (**B**) PcoA analysis; (**C**) Species Venn diagram; (**D**) Community bar chart at the phylum classification level; (**E**) Community bar chart at the genus classification level; (**F**) Prediction of COG function in gut microbiota; (**G**) Functional prediction at KEGG level 1; (**H**). Functional prediction at KEGG level 2; (**I**) Functional prediction at KEGG level 3.

To compare the species richness and diversity of gut microbial communities among BSB, MC, and BM, we calculated six alpha diversity indices: Sobs, Shannon, Ace, Chao, Coverage, and Heip. Differences in alpha diversity among the three groups (BSB, MC, BM) were tested using the Kruskal-Wallis test, followed by Dunn’s post-hoc test for pairwise comparisons. As summarized in Table 4, none of the indices showed significant differences (*p* > 0.05 for all comparisons). These results indicate that the three fish species harbor gut microbial communities with comparable richness, evenness, and overall diversity. The lack of significant differences in alpha diversity suggests that the distinct feeding habits among BSB, BM, and MC primarily influence community composition (beta diversity) rather than within-sample diversity. Beta diversity analysis at the phylum level is shown in Figure 5A. BSB, MC, and BM each formed distinct clusters, with BSB and BM grouping together into a common branch. This indicates that the gut microbial communities of BSB and BM are more similar, and that differences in microbiota composition are closely associated with the feeding habits of each species. Analysis of operational taxonomic units (OTUs) revealed that BSB, MC, and BM shared 204 OTUs, accounting for 7.29% of the total. BM shared 466 OTUs with its maternal parent BSB (16.65%) and 256 OTUs with its paternal parent MC (9.15%) (Figure 5B,C). Thus, the gut microbial community of BM showed significantly higher similarity (*p* < 0.05) to that of BSB than to MC. After filtering out chloroplast and mitochondrial sequences, the dominant bacterial phyla in all three fish species were Fusobacteriota, Proteobacteria, Firmicutes, and Chloroflexi. The relative abundance of Fusobacteriota was significantly higher in BSB and BM than in MC. Cyanobacteria were present at low abundance (<1.5%) in all groups, with no significant differences among BSB, MC, and BM (Figure 5D,E).

Functional prediction of the gut microbiota indicated that the main microbial functions were related to metabolism, genetic information processing, environmental information processing, and cellular processes. Analysis of COG functional abundance showed that in carbohydrate transport and metabolism, BM and BSB were significantly higher than MC (*p* < 0.05). Conversely, in lipid transport and metabolism, MC was significantly higher than both BM and BSB (*p* < 0.05) (Figure 5F). The functions of the gut microbiota were predicted at the three KEGG levels (Figure 5G–I). At KEGG level 1, 75% of the gut microbiota was associated with metabolism, and 6.9% was associated with genetic information processing (Figure 5G). At KEGG level 2, the gut microbiota was primarily involved in pathways such as carbohydrate metabolism and amino acid metabolism (Figure 5H). Finally, at KEGG level 3, the enriched pathways included Ribosome, Purine metabolism, and amino sugar and nucleotide sugar metabolism (Figure 5I).

### 2.7. qRT-PCR Validation

**Figure 6:**
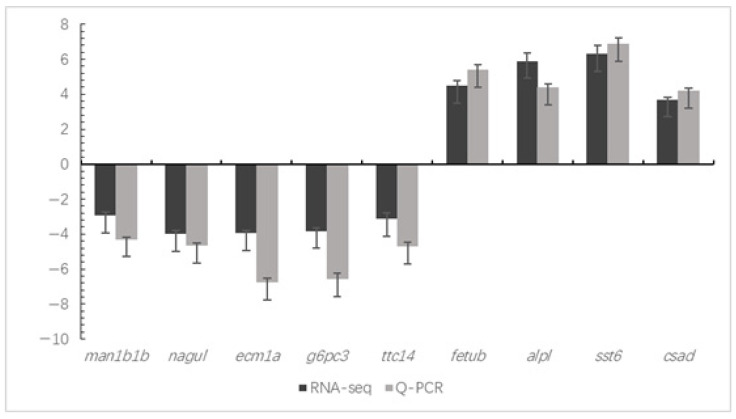
qPCR Validation of differentially expressed genes.

The qRT-PCR validation of 10 genes is displayed in Figure 6. The expression trends of different groups are consistent with the transcriptome data, indicating that the RNA-sequencing results are reliable.

## 3. Discussion

In 2022, global aquatic animal production reached a historic high of 185 million tons, with aquaculture contributing 94 million tons (51%), surpassing capture fisheries for the first time [17]. With the expansion of aquaculture, the demand for high-quality, high-yield species has become increasingly urgent. Hybridization is a powerful breeding tool that can combine desirable traits and generate heterosis in growth [26,27], disease and stress resistance [28,29], and flesh quality [30]. Moreover, hybridization can also modify feeding habits, such as crossing carnivorous fish with herbivorous fish to produce offspring with a tendency toward herbivory. This can reduce the reliance on high-protein feed during farming, thereby lowering production costs.

In previous studies, we successfully obtained a new diploid hybrid offspring BM with a high hatching rate, by crossing the herbivorous BSB with the carnivorous MC. Preliminary research results indicate that BM has a tendency toward herbivorous feeding habits and exhibits greater growth advantages than both parents [31]. However, the adaptive changes and genetic basis underlying the feeding habit transformation in BM hybrid fish require further investigation. In this study, we systematically compared BM with its parents in terms of feeding organ morphology, digestive enzyme activities, liver transcriptome, and gut microbiota. The results provide multi-dimensional evidence for BM’s herbivorous adaptation and improved growth potential.

### 3.1. Morphological Evidence: Intermediate Intestinal Traits Inherited from the Herbivorous Parent

The morphological characteristics of fish are adapted to their feeding strategies. Hybrid offspring typically display three inheritance patterns: paternal-dominant, intermediate, or maternal-dominant [32]. For example, hybrid offspring from *Channa maculata* (♀) × *Channa argus* (♂) exhibit distinct paternal morphological traits [33]; the natural gynogenetic offspring from *Megalobrama amblycephala* (♀) × *Siniperca chuatsi, common carp* (♀) × *topmouth culter* (♂) display morphological features resembling those of the maternal parent [34,35]; and hybrid offspring from *Schizothorax waltoni* (♀) × *Schizothorax oconnori* (♂) exhibit intermediate morphological traits [36].In our study, BM showed intermediate but maternally biased morphological features.

Specifically, BM had significantly smaller ML/MH, HL/HH, and MH/HH ratios than MC but larger ratios than BSB (Table 1, *p* < 0.05). Compared to the carnivorous fish MC, BM has a shorter and smaller head with a slightly upturned lower jaw (Figure 1 and Table 1), which was more advantageous for feeding on herbivorous plants and algae [37]. Regarding the intestine, BM exhibited more folds (5–6) than MC (2–3), approximating BSB (7–8) (Figure 1). The relative gut length (RGL) of BM was 1.90, intermediate between BSB (2.24) and MC (0.97) (Table 1), which fits the general rule that herbivores have RGL 2–5 and carnivores RGL < 1 [38]. Histologically, BM’s villus width (113.0 μm) was not significantly different from BSB (115.2 μm, *p* > 0.05) but was significantly greater than that of MC (86.7 μm, *p* < 0.05) (Figure 2 and Table 2). This quantitative evidence indicates that BM has enhanced absorptive surface area compared to MC, a feature that would facilitate more efficient digestion of plant-based diets [16,39]. Together, these morphological and histological data provide strong support for BM’s herbivorous tendency.

### 3.2. Digestive Enzyme Activities: Functional Shift Toward Carbohydrate Digestion

Digestive enzyme activities reflect nutrient absorption and digestion capacity. Carnivorous fish generally have higher protease and lipase, while herbivorous fish possess higher amylase and cellulase [3,4]. Our enzyme activity measurements revealed that BM’s α-amylase activity was significantly higher than that of MC (*p* < 0.05) and significantly lower than that of BSB (*p* < 0.05) in both intestine and liver (Figure 3A). Conversely, trypsin and lipase activities in BM were significantly lower than in MC (*p* < 0.05) but higher than in BSB (*p* < 0.05) (Figure 3B,C). Thus, BM displayed an intermediate digestive enzyme profile functionally shifted toward carbohydrate digestion relative to the carnivorous parent. This “generalist” digestive configuration may allow BM to utilize both plant and animal food sources flexibly, potentially explaining its superior growth [21]. Similar patterns have been observed in hybrids of *Mastacembelus armatus* (♀) × *M. favus* (♂) [40] and *Percocypris pingi* (♀) × *Schizothorax wangchiachii* (♂) [41], supporting the general applicability of hybridization for modifying digestive physiology.

### 3.3. Muscle Composition: Improved Protein Content and Reduced Fat

The protein content in fish meat is a key factor in evaluating fillet quality. Our proximate composition analysis showed that BM had a crude protein content of 19.63%, which was significantly higher than both BSB (18.50%) and MC (18.30%) (*p* < 0.05). Meanwhile, crude fat content in BM (1.60%) was significantly lower than in MC (2.33%, *p* < 0.05) and similar to BSB (1.47%, *p* > 0.05) (Table 3). This combination of higher protein and lower fat is highly desirable for aquaculture products. Similar improvements in meat quality have been reported in other hybrid combinations, including Japanese white crucian carp (♀) × red crucian carp (♂) [42], largemouth bass (♀) × green sunfish (♂) [24], and grass carp (♀) × topmouth culter (♂) [10]. The enhanced protein content in BM may be linked to its more efficient nutrient utilization from plant-based feeds, as suggested by the digestive enzyme and transcriptome data.

### 3.4. Transcriptomic Evidence: Key Differentially Expressed Genes and Maternal Expression Bias

The liver plays a central role in metabolic homeostasis and nutrient processing, making it an ideal tissue for studying transcriptional responses associated with feeding habits [43]. We identified a total of 10,423 DEGs between MC and BSB, 7980 DEGs between BM and MC, and 5247 DEGs between BM and BSB (Figure 4A). The higher number of DEGs between BM and MC compared to BM and BSB suggests that BM’s liver transcriptome is more similar to that of the herbivorous parent (BSB), consistent with the morphological and enzymatic data.

Several key genes involved in gluconeogenesis and glycolysis showed differential expression patterns consistent with herbivorous adaptation. The *g6pc3* (glucose-6-phosphatase catalytic subunit 3), which catalyzes the final step of gluconeogenesis, was upregulated 3.2-fold in BM compared to MC. Similarly, *pck1* (phosphoenolpyruvate carboxykinase 1), another rate-limiting enzyme in gluconeogenesis, was upregulated 2.8-fold in BM vs. MC. These upregulations suggest that BM has a greater capacity for endogenous glucose production, a feature often observed in herbivorous fish that need to maintain blood glucose levels on carbohydrate-rich diets [44]. In contrast, *gck* (glucokinase), which facilitates glucose utilization, showed no significant difference between BM and MC, indicating that BM is not simply “more herbivorous” but has a distinct metabolic configuration that combines enhanced glucose production with maintained utilization capacity.

The peroxisome proliferator-activated receptor gamma (*pparγ*), a master regulator of adipogenesis and lipid storage, was downregulated 2.1-fold in BM compared to MC; *fasn* (fatty acid synthase), which catalyzes de novo lipogenesis, was also downregulated 1.9-fold in BM vs. MC. These downregulations align with the significantly lower crude fat content in BM (1.60%) compared to MC (2.33%) (Table 3). Conversely, *cpt1a* (carnitine palmitoyltransferase 1A), which controls fatty acid oxidation, showed no significant difference, suggesting that reduced lipid accumulation in BM is primarily due to decreased synthesis rather than increased oxidation. The *arg1* (arginase 1), which catalyzes the final step of the urea cycle and is involved in protein catabolism, was downregulated 2.5-fold in BM compared to MC. This suggests lower protein catabolism in BM, which is consistent with herbivorous fish deriving more energy from carbohydrates rather than proteins [4]. The *got1* (glutamic-oxaloacetic transaminase 1), which is involved in amino acid degradation, showed a similar downregulation pattern (1.8-fold).

Taken together, the transcriptomic data provide molecular evidence that BM has acquired a herbivore-like metabolic program, particularly in carbohydrate and lipid metabolism, with a strong maternal inheritance pattern. Expression bias analysis revealed that 68.2% of biased genes in BM favored BSB. These findings complement the morphological, enzymatic, and microbiota data in supporting BM’s herbivorous tendency.

### 3.5. Gut Microbiota: Similarity to Herbivorous Parent After Proper Filtering

The intestinal microbiota of fish plays a crucial role in the immune and digestive systems [45]. Changes in microbiota diversity and structure are primarily driven by feeding habits [46,47] and environment [48], and the microbiota significantly impacts fish growth, health, and ecological adaptation.

After filtering out chloroplast and mitochondrial sequences, the dominant bacterial phyla in all three fish groups were Fusobacteriota, Proteobacteria, Firmicutes, and Chloroflexi (Figure 5D). The relative abundance of Fusobacteriota was significantly higher in BSB (45.3 ± 18.1%) and BM (46.2 ± 12.2%) than in MC (0.2 ± 0.1%) (*p* < 0.05). This result is similar to studies in grass carp [49], where Fusobacteriota were dominant in herbivorous fish. True Cyanobacteria (autotrophic bacteria) were present at low abundance (<1.5%) in all groups, with no significant differences among BSB, BM, and MC.

At the genus level, Cetobacterium was dominant in BSB (45.3%) and BM (46.2%), while MC showed a more diverse composition with Cetobacterium (0.2%) (Figure 5E). Cetobacterium has been reported to play an important role in sugar absorption and metabolism [50], so its higher abundance in BSB and BM is consistent with their greater carbohydrate digestion capacity (Figure 3A). PICRUSt2 functional prediction indicated that BSB and BM had significantly higher levels of predicted carbohydrate transport and metabolism compared to MC (*p* < 0.05), but lower levels of lipid transport and metabolism (*p* < 0.05) (Figure 5G–I). These predictions are consistent with our direct measurements of digestive enzyme activities, providing cross-validation.

Beta diversity analysis showed that BSB and BM clustered together, separate from MC (Figure 5B), indicating that the gut microbial community composition of BM is more similar to its herbivorous parent. This pattern aligns with the vertical transmission hypothesis: BM shares its rearing environment and maternal lineage with BSB, which likely shapes its microbiota toward a herbivorous-like profile.

### 3.6. Integrative Perspective and Limitations

The concordance across multiple levels provides compelling evidence that BM exhibits a herbivorous tendency. However, we acknowledge several limitations. First, all fish were fed a single artificial diet; therefore, direct behavioral choice experiments with plant versus animal diets would be needed to definitively establish feeding preference. Second, our study focused on adult fish; ontogenetic changes in feeding habits during early development remain unknown. Third, PICRUSt2-based functional predictions have inherent limitations; metabolomics would provide higher-resolution evidence. Fourth, the liver transcriptome provides correlative rather than causal evidence; functional validation would strengthen mechanistic conclusions. Future studies should address these limitations and explore the developmental trajectory of feeding habit formation in BM.

An interesting observation in this study is that the gut microbiota of BM showed higher similarity to the herbivorous parent BSB, whereas the liver transcriptome of BM was clearly separated from both parents (Figure 4B). This apparent discordance likely reflects different temporal and mechanistic scales of adaptation. Gut microbiota is highly plastic and responds rapidly to dietary changes (within days to weeks); it is also strongly influenced by vertical transmission (from the mother) and environmental sharing. BM shares its rearing environment and maternal lineage with BSB, which explains why its microbiota resembles BSB more closely. In contrast, liver gene expression represents a more integrated, genetically-programmed metabolic state that reflects the hybrid genome. BM’s transcriptome is clearly distinct from both parents, with 68.2% of biased genes favoring BSB but still showing unique hybrid expression patterns. This indicates that while BM harbors a ‘herbivorous-like’ microbial community (likely beneficial for plant digestion), its own metabolic program is not fully herbivorous but rather a complementary hybrid of both parental capacities. This complementarity, a herbivorous-like microbiota plus an intermediate/heterotic transcriptome, may contribute to BM’s superior growth (heterosis) compared to either parent. Future studies using gnotobiotic experiments or microbiota transplantation could help determine whether the microbiota drives host gene expression changes or vice versa.

From an evolutionary perspective, both parental species belong to the family Cyprinidae, which is widely considered to have originated from omnivorous or herbivorous ancestors in East Asian freshwater ecosystems during the Eocene–Oligocene transition; its members have since diversified into a wide range of trophic niches [51,52]. BSB has retained and specialized toward herbivory, as evidenced by its long intestine, high amylase activity, and Cetobacterium-dominated gut microbiota. In contrast, MC has secondarily evolved toward carnivory, a derived trait within cyprinids. When the two genomes are combined in the hybrid BM, the offspring does not merely average the parental phenotypes. Instead, BM exhibits a maternally biased reversion toward the ancestral herbivorous state. This interpretation is supported by our multi-dimensional results, including maternal bias in liver gene expression (68.2% of biased genes favor BSB), intermediate but BSB-like relative gut length, and a gut microbiota composition that clusters with the herbivorous parent BSB. We speculate that the carnivorous adaptations in MC may be genetically recessive or easily overridden by the more deeply conserved herbivorous regulatory networks inherited from BSB. The upregulation of gluconeogenic genes (*g6pc3*, *pck1*) and downregulation of lipogenic genes (*pparγ*, *fasn*) in BM compared to MC further support the reactivation of a herbivore-like metabolic program.

From an ecological perspective, BM displays an intermediate or generalist phenotype that lies between a specialist herbivore (BSB) and a specialist carnivore (MC). Generalist feeders typically possess greater ecological plasticity, enabling them to exploit variable food resources. In aquaculture, this is highly advantageous. BM can utilize plant-based feeds more efficiently than MC (thereby reducing reliance on high-protein fishmeal and lowering production costs), while still retaining some digestive capacity for animal protein. This dual capability may explain BM‘s superior growth compared to both parents (heterosis), as it can better adapt to the single formulated diet provided in the rearing environment. From a broader ecological perspective, such hybrid generalists could have a competitive advantage in fluctuating or resource-limited environments, where dietary flexibility enhances survival and growth. Collectively, BM’s herbivorous tendency is not merely a simple hybrid intermediate but rather an evolutionarily re-emerged and ecologically beneficial generalist strategy.

## 4. Materials and Methods

### 4.1. Ethics Statement

According to the regulations issued by the Administration of Affairs Concerning Animal Experimentation, prior authorization from the Science and Technology Bureau of China or the Department of Wildlife Administration is not required for studies involving fish species that are not listed as rare or endangered (first- or second-class state protection levels). All experimental procedures in this study were reviewed and approved by the Biomedical Research Ethics Committee of Hunan Normal University (approval number: 2023 No. (610)). Before dissection, fish were deeply anesthetized using MS-222 at a concentration of 100 mg/L (Sigma-Aldrich, St. Louis, MO, USA). All personnel involved in animal care and experimental procedures had completed professional training and were certified by the Institute of Experimental Animals, Hunan Province, China.

### 4.2. Experimental Fish Sources

The MC, BSB, and BM were obtained from the Engineering Research Center of Polyploid Fish Reproduction and Breeding of the State Education Ministry, Hunan Normal University. Three to five days post-hatching, BSB, MC, and BM were transferred to different ponds at a density of 150,000 fry/667 m^2^. The fry were fed plankton and 3–5 L of soybean milk daily. After almost a month, three kinds of experimental fish were randomly selected and stocked at a density of 1500 fish/667 m^2^ (500 fish for each kind) for the adult grow-out stage from 1 month to 2 years post-hatching, and were fed a commercial floating feed (crude protein > 40%) twice a day at 3% body weight. This uniform diet allowed us to compare genetic differences in digestion and metabolism without confounding by diet variation. Water quality analysis was performed using a multi-parameter water quality meter (YSI, Yellow Springs, OH, USA). Water quality parameters were maintained at pH between 7 and 8.5, ammonia and nitrite concentrations were lower than 0.01 mg/L, and dissolved oxygen was higher than 5 mg/mL.

### 4.3. Comparison of Structural Characteristics of Feeding Organs

Twenty individuals from each group (BSB (676.5 ± 96 g), MC (600.3 ± 71 g), and BM (634.7 ± 67 g) were randomly selected for morphological examination. The examined measurable traits included mouth width, mouth height, head length, head height, body length, and intestine length. The following ratios were calculated based on the measured data: mouth width/mouth height (ML/MH), head length/head height (HL/HH), mouth height/head height (MH/HH), body length/mouth width (BL/MW), and intestine length/body length (IL/BL).

### 4.4. Intestinal Histology and Digestive Enzyme Activity Assay

Fish were fasted for 24 h before sampling, and the muscle, liver, and intestine were sampled. The intestine of the fish was divided into three parts: anterior intestine, middle intestine, and posterior intestine [5]. Intestinal tissues were excised and photographed. Tissue samples measuring approximately 0.5–1 cm in length were collected from the anterior, middle, and posterior segments of the intestine. The samples were immediately fixed in 4% paraformaldehyde for 24 h. Standard paraffin sections (6 μm thickness) were prepared and stained with hematoxylin and eosin (H&E). Histological structures of the intestine were examined and photographed under a light microscope. Image analysis was conducted using ImageJ software of windows version 1.54p (National Institutes of Health, Bethesda, MD, USA). Morphometric parameters assessed included intestinal wall thickness, circular muscle layer thickness, longitudinal muscle layer thickness, villus height, and villus width. For each intestinal segment, three to five midgut cross-sectional images were randomly selected for measurement. In each section, five microscopic fields were analyzed, and the average value of each parameter was calculated and recorded as the representative value for each individual. The liver and intestinal tissues were carefully collected to measure enzyme activities, including α-amylase, pepsin, trypsin, and lipase. The sample preparation and assay procedures followed the instructions in the commercial kits (Nanjing Jiancheng Bioengineering Institute), with six replicates per fish group. The remaining liver tissue was sampled for transcriptome sequencing; after removing tissue sections for enzyme activity assays, the remaining intestinal tissues were fixed in 4% paraformaldehyde for histological analysis. For gut microbiota analysis, the intestinal contents were collected by gently squeezing the intestine with sterile forceps, transferred to sterile cryotubes, immediately frozen in liquid nitrogen, and stored at −80 °C until DNA extraction.

### 4.5. Proximate Composition in Back Muscle

The crude protein content in the muscle was determined using the Kjeldahl method (GB/T 6432-2018) [53], the fat content was measured by acid hydrolysis (GB 5009.6-2016) [54], and the moisture and ash contents were determined according to the methods specified in GB 5009.3-2016 [55] and GB 5009.4-2016 [56], respectively.

### 4.6. Sequencing and Analysis of Transcriptome

RNA extraction was performed from the liver using the standard Trizol method. The OD value of high-quality RNA ranged from 2.0 to 2.2, and the RIN value was no lower than 7. There are three sets of liver RNA for each group (BSB, MC, and BM). Each set of RNA consisted of pooled RNA extracted from the liver tissue of three different individuals. cDNA libraries were constructed using the NEBNext Ultra RNA Library Prep Kit for Illumina (NEB, Ipswich, MA, USA) following the manufacturer’s instructions. Briefly, mRNA was enriched from 1 μg of total RNA using poly-T oligo-attached magnetic beads, then fragmented, reverse transcribed into cDNA, end-repaired, A-tailed, and ligated with Illumina adapters. The final libraries were amplified by PCR and validated for quality and concentration. Qualified libraries were sequenced on the Illumina HiSeq 2500 platform with paired-end 150 bp (PE150) read length. The average sequencing depth was 21.4 Gb per sample (range: 21.34 Gb for MC, 21.75 Gb for BM, 23.13 Gb for BSB). Raw reads were processed using fastp (v0.20.0) to remove adapter sequences, filter out reads containing more than 10% unknown bases (N), and discard low-quality reads (Q-score < 20). The resulting clean reads were used for all subsequent analyses. Clean reads were aligned to the reference genome of *Megalobrama amblycephala* (GCF_018677685.1) using STAR (v2.7.9a) with default parameters. Gene expression levels were quantified as transcripts per million (TPM) and raw read counts using RSEM (v1.3.3). Differential expression analysis was performed using DESeq2 (v1.34.0) in R. Genes with an adjusted *p*-value (Padj) < 0.001 (Benjamini-Hochberg correction) and an absolute log2 fold change (|log2FC|) ≥ 1 were considered significantly differentially expressed. Genes were classified into two expression patterns according to the following criteria: genes showing no significant difference in expression between BM and BSB, but a significant difference compared to MC were categorized as maternally biased, whereas genes with no significant difference between BM and MC but a significant difference from BSB were classified as paternally biased.

### 4.7. Sequencing and Analysis of Intestinal Microbiota

Total genomic DNA was extracted from intestinal content samples using the QlAamp DNA Kit (Qiagen, Hilden, Germany) following the manufacturer’s protocol. DNA concentration and purity were measured using a NanoDrop 2000 spectrophotometer (Thermo Scientific, Waltham, MA, USA). The extracted DNA of the intestinal microbiota was employed for the amplification of the specified regions. The primer sequences are 338-F (5′-ACTCCTACGGGGAGGCAGCAG-3′) and 806-R (5′-GGACTACHVGGGTWTCTAAT-3′). PCR products were purified and sequenced on an Illumina NovaSeq 6000 platform (2 × 250 bp). Raw reads were quality-filtered and denoised using DADA2 in QIIME2. Operational taxonomic units (OTUs) were clustered at 97% similarity and taxonomically assigned against the Silva 138 database. OTUs classified as chloroplast or mitochondria were removed. Alpha diversity indices (Sobs, Shannon, Ace, Chao, Coverage, Heip) were calculated, and differences among groups were tested using Kruskal-Wallis test. Beta diversity was assessed using Bray-Curtis dissimilarity and visualized via PCoA and UPGMA clustering. Functional potential was predicted using PICRUSt2, with KEGG and COG annotations. Limitations of PICRUSt2 are noted. PICRUSt2 has known limitations as it infers functional potential from 16S rRNA data rather than direct metagenomic or metabolomic measurements; therefore, these results are considered exploratory and hypothesis-generating.

### 4.8. qRT-PCR Validation

To validate the accuracy of the transcription data, the expression of 10 genes was examined by quantitative reverse transcription polymerase chain reaction (qRT-PCR). The primer sequences are presented in Table A1. qRT-PCR was performed using SYBR Green Master Mix (Takara, Kusatsu, Japan) on a LightCycler 480 system (Roche, Mannheim, Germany). Reaction conditions: 95 °C for 30 s, followed by 40 cycles of 95 °C for 5 s and 60 °C for 30 s. Each group contained three biological replicates, each with three technical replicates. β-actin was used as the internal control. Relative expression was calculated using the 2^−ΔΔCt^ method.

### 4.9. Statistical Analysis

All data were presented as mean ± standard deviation (SD). Normality was checked using the Shapiro–Wilk test; homogeneity of variances was checked using Levene’s test. For comparisons among three groups (BSB, MC, BM), one-way ANOVA followed by Tukey’s HSD post-hoc test was used. For comparisons between two groups, a two-tailed Student’s *t*-test was applied. For transcriptome DEGs, DESeq2 used the Wald test with Benjamini–Hochberg FDR correction (Padj < 0.001, |log2FC| ≥ 1). For beta diversity, PERMANOVA (adonis, 999 permutations) was used. Statistical significance was set at *p* < 0.05. All analyses were performed in R v4.2.0.

## 5. Conclusions

This study provides multi-dimensional evidence that the hybrid fish BM, derived from herbivorous female *M. amblycephala* and carnivorous male *C. mongolicus*, exhibits a predominantly herbivorous feeding adaptation with superior growth and muscle quality traits. Morphologically, BM shows intermediate RGL (1.90) and intestinal fold numbers (5–6) between BSB (2.24, 7–8) and MC (0.97, 2–3), with quantitative histology confirming enhanced absorptive surface area compared to MC. Digestive enzyme activities in BM are intermediate, α-amylase is significantly higher than in MC (*p* < 0.05), and trypsin and lipase are significantly lower than in MC (*p* < 0.05), supporting enhanced carbohydrate digestion capacity. The muscle composition of BM shows significantly higher crude protein (19.63%) than both parents (18.3–18.5%, *p* < 0.05) and lower crude fat (1.60%) than MC (2.33%, *p* < 0.05), indicating improved fillet quality. Liver transcriptome reveals that BM’s gene expression is biased toward the maternal BSB (68.2%) with enriched pathways related to carbohydrate and lipid metabolism, consistent with herbivorous adaptation. Gut microbiota of BM is more similar to BSB than to MC, with Cetobacterium dominance and enhanced predicted carbohydrate metabolism functions. BM reduces reliance on high-protein feeds (cost reduction of 20–30%), produces higher-protein meat, and shows faster growth than both parents, making it a promising candidate for sustainable aquaculture. The reproducible hybridization protocol (fertile diploid, self-crossing possible) ensures commercial scalability. Future research should focus on early developmental stages, direct feeding preference tests, and metabolomic validation.

## Figures and Tables

**Table 1:** Comparison of the measurable traits among MC, BSB, and BM.

Groups	MC	BSB	BM
ML/MH	1.14 ± 0.02 ^a^	0.95 ± 0.10 ^c^	1.00 ± 0.02 ^b^
HL/HH	1.70 ± 0.03 ^a^	1.15 ± 0.01 ^c^	1.32 ± 0.02 ^b^
MH/HH	0.87 ± 0.04 ^a^	0.56 ± 0.03 ^c^	0.61 ± 0.03 ^b^
BL/MW	8.85 ± 0.46 ^c^	13.05 ± 0.36 ^a^	12.48 ± 0.43 ^b^
IL/BL	0.97 ± 0.21 ^c^	2.24 ± 0.14 ^a^	1.90 ± 0.12 ^b^

Note: Different letters in the same row indicate significant differences (*p* < 0.05).

**Table 2:** Mid-intestinal related indices statistics in BSB, MC, and hybrid offspring BM.

Fish	VH (μm)	VW (μm)	CM (μm)	LM (μm)	MW (μm)
BSB	1064.1 ± 119.3 ^c^	115.2 ± 14.9 ^a^	118.8 ± 24.1 ^a^	61.7 ± 9.2	197.0± 35.8 ^a^
BM	1210.0 ± 92.7 ^ᵇ^	113.0 ± 15.6 ^a^	115.8 ± 14.1 ^a^	69.1 ± 16.9	202.0 ± 31.8 ^a^
MC	1350.0 ± 120.2 ^a^	86.7 ± 7.7 ^b^	88.1 ± 12.0 ^b^	69.4 ± 18.7	156.5± 35.8 ^b^

Note: Different letters in the same row indicate significant differences (*p* < 0.05).

**Table 3:** Proximate composition of back muscles in MC, BSB, and BM.

Nutritional Composition	MC (g/100 g)	BSB (g/100 g)	BM (g/100 g)
Crude protein	18.30 ± 0.10 ^b^	18.50 ± 0.20 ^b^	19.63 ± 0.15 ^a^
Crude fat	2.33 ± 0.25 ^a^	1.47 ± 0.15 ^b^	1.60 ± 0.26 ^b^
Moisture	77.80 ± 0.26 ^b^	78.90 ± 0.36 ^a^	77.30 ± 0.35 ^b^
Ash	1.00 ± 0.09 ^a^	1.13 ± 0.06 ^a^	1.10 ± 0.05 ^a^

Note: Different letters in the same row indicate significant differences (*p* < 0.05).

**Table 4:** Alpha diversity indices of gut microbiota for BSB, MC, and BM (mean ± SD, n = 6 per group).

Index	BSB	MC	BM	*p*-Value (Kruskal-Wallis)
Sobs	198.4 ± 15.2	192.6 ± 14.8	196.3 ± 16.1	0.52
Shannon	3.42 ± 0.31	3.35 ± 0.33	3.38 ± 0.28	0.67
Ace	242.3 ± 17.5	236.8 ± 18.2	239.5 ± 16.7	0.61
Chao	245.6 ± 18.3	238.7 ± 19.5	241.2 ± 16.9	0.58
Coverage	0.998 ± 0.001	0.998 ± 0.001	0.998 ± 0.001	0.89
Heip	0.52 ± 0.06	0.50 ± 0.07	0.51 ± 0.05	0.71

## Data Availability

The complete clean reads for the libraries used in this study have been uploaded to the NCBI Sequence Read Archive (SRA) site (http://www.ncbi.nlm.nih.gov/sra/ (accessed on 9 February 2026); BioProject ID PRJNA1421132).

## Appendix A

**Table A1:** Primers for the validation of RNA sequencing.

Gene Name	Primer (5′-3′)	Application
*man1b1b*	F: CACAGCTGGAGGCTCGAA	qRT–PCR
	R: GGACAACTGTTTCCATTTCCTCC	qRT–PCR
*naglu*	F: TTCCAGCGAATTGGAGCACT	qRT–PCR
	R: TTGCTTGAGGGTCCACTGAC	qRT–PCR
*ecm1a*	F: CTGTGCGGAAAAAGCATGGAA	qRT–PCR
	R: AGGGGTTGGTAGAAGGGGTT	qRT–PCR
*g6pc3*	F: ACACTCGCACAGGCAGTAAG	qRT–PCR
	R: GAGGGAAGTGAGCCAGGATG	qRT–PCR
*ttc14*	F: TCTTCATTATACCCGGGGCG	qRT–PCR
	R: GGTTGGACAAACCAAGCGAG	qRT–PCR
*fetub*	F: CAGCAGCACAACAGGCTCGTC	qRT–PCR
	R: CAGGGAAGGATGGAAGGGTC	qRT–PCR
*alpl*	F: TCTTGGTGATGGTATGGGTGTA	qRT–PCR
	R: CCACTGATTTGCCTGCGTCT	qRT–PCR
*sst6*	F: ACCTCACAAACAATAATCAGCTC	qRT–PCR
	R: CAGGAGGATGGCGTGATTGT	qRT–PCR
*csad*	F: TGTTCCTTACTGAGGCTTTC	qRT–PCR
	R: GGAGCCACTTCATAGGTGTA	qRT–PCR
*β-actin*	F: CCTGTTGGCTTTGGGATTGA	qRT–PCR
	R: TCTACAACGAGCTGCGTGTTG	qRT–PCR

**Table A2:** Quality assessment of RNA sequencing data.

Samples	Raw Reads	Clean Reads	Clean Bases	Error (%)	Q20 (%)	Q30 (%)	GC Content (%)
MC	143,703,794	142,766,990	21.34 G	0.0117	98.90	96.54	48.72
BM	145,697,564	144,834,792	21.75 G	0.0117	98.91	96.53	48.78
BSB	155,411,310	154,509,122	23.13 G	0.0117	98.94	96.64	49.19
